# Assessment of oral appliance for obstructive sleep apnea patients

**DOI:** 10.1002/cre2.35

**Published:** 2016-07-29

**Authors:** Eri Makihara, Toshihiro Kawano, Ryuichiro Miyajima, Shin‐ichi Masumi, Reyes Enciso, Glenn T. Clark

**Affiliations:** ^1^ Division of Occlusion & Maxillofacial Reconstruction, Department of Oral Function, School of Dentistry Kyushu Dental University Kitakyushu Japan; ^2^ Division of Dental Public Health and Pediatric Dentistry, Ostrow School of Dentistry of USC University of Southern California Los Angeles CA USA; ^3^ Division of Diagnostic Sciences, Ostrow School of Dentistry of USC University of Southern California Los Angeles CA USA

**Keywords:** Obstructive sleep apnea, oral appliances, questionnaire survey

## Abstract

Although oral appliances (OAs) have become widely used for the management of obstructive sleep apnea (OSA), side effects of OAs are generally related to poor utilization. The purpose of the present study was to evaluate relationship between utilization and treatment efficacy of a boil‐and‐bite appliance for OSA patients. A total of 135 patients with OSA who had used an OAs were mailed a questionnaire to determine whether they were currently using the OA. If so, they were asked about OA use, improvement of signs and subjective symptoms, and utilization. Otherwise, they were asked to indicate why and when they quit using the OA. Results of overnight polysomnography (PSG) before and after treatment were reviewed. Of the 48 responding patients, 33 patients were currently using the OA. The most common complication was excessive salivation (*n* = 11). All indices from PSG excluding arousal index were significantly improved after treatment (*p* < 0.05). Thirty patients showed improved signs and subjective symptoms. Eight out of 12 subjects (66.7%) were successfully treated, achieving an apnea‐hypopnea index (AHI) < 10/h and >50% reduction in apnea‐hypopnea index. Of the 15 patients no longer using the OA, the primary reason for quitting was “no treatment effect” (*n* = 5). No indices from PSG recording differed between before and after treatment in the not‐using group. These results suggest that both subjective and objective signs and symptoms improved with use of the OA in the using group. However, no signs and subjective symptoms or indices of sleep quality differed between before and after treatment in the not‐using group. Device improvements are needed to achieve better treatment efficacy, and thus improve compliance. The present study evaluated relationship between utilization and treatment efficacy of a boil ‐and bite appliance for OSA patients. Device improvements are needed to achieve better treatment efficacy, thus improve compliance.

## Introduction

Obstructive sleep apnea (OSA) is caused by partial or complete obstruction of the upper airway during sleep (Clark et al. 2000). Continuous positive airway pressure is the gold standard of treatment, but despite its effectiveness, compliance rates have declined because the required systems are noisy and wearing the mask can cause discomfort in some users (Cistulli et al. 2004). Use of oral appliances (OAs) is indicated for patients with mild‐to‐moderate OSA. The aim of OA use is to advance the mandible slightly to enlarge the upper airway and prevent collapse during sleep. OAs improve daytime symptoms, cardiovascular and neurocognitive functions, and quality of life (Hoffstein 2007). Although numerous OAs with various designs have been claimed to help in managing OSA (American Sleep Disorders Association report 1995), side effects such as dry mouth, excessive salivation, tooth discomfort, occlusal changes, muscle tenderness, and jaw stiffness have been reported in association with this approach (Almeida et al. 2006).

Oral appliances use of either a one‐piece, non‐titratable design (monobloc), or a two‐piece, titratable design (bibloc) are either custom‐made or non‐custom‐made (Ramar et al. 2015). A non–custom OA generally requires only an individual molding of a thermoplastic material, while a custom OA usually requires dental impressions, bite registration, and fabrication by a dental laboratory. The one‐piece design fixes the mandible rigidly in an anterior position, whereas the two‐piece design usually allows some freedom of mandibular movement (i.e., lateral, vertical, and/or anterior) (Hoekema et al. 2004), which has been suggested to decrease the chance of temporomandibular disorders and improve patient comfort (Henke et al. 2000).

Discontinuation of OA treatment is generally related to side effects, complication, or a lack of perceived benefits (McGown et al. 2001). Some studies have observed similar frequencies of side effects in using and not‐using patients, whereas others have reported a higher number of side effects in patients who discontinued treatment (Clark et al. 2000; McGown et al. 2001). Thus, improvements in side effects might increase the number of patients who can continue to use the OA for a long time.

The purpose of the present study was to investigate relationship between the usability and treatment efficacy of a boil‐and‐bite appliance with the 50% anterior mandibular position as the initial position, representing a non‐custom, semi‐titratable OA for OSA patients.

## Materials and Methods

### Subjects

A total of 135 patients who had been referred by various sleep physicians to Kyushu Dental University in Japan from January 2006 to July 2013 for treatment of OSA with an OA were included in this study. Inclusion criteria were a diagnosis of OSA with no other sleep disorders based on overnight polysomnography (PSG) lasting at least 5 h, and performed at one of 13 medical institutions around Kitakyushu City. Before treatment, a clinical examination of the stomatognathic system was performed, including measurement of mandibular mobility, palpation of the temporomandibular joints and masticatory muscles, and recording of pain during jaw motion were performed (Dworkin and LeResche 1992). Any subjects exhibiting signs and symptoms related to temporomandibular joint dysfunction, a history of psychological problems, or occlusion dysfunction was excluded from this study.

All study protocols that were conducted in full accordance with the Declaration of Helsinki, were approved by the ethics committee at Kyushu Dental University (approval number: 12‐17; approved 31 October 2012). All patients read and signed an informed consent.

### Oral appliance

All patients used a TheraSnore^TM^ appliance (DISTAR, Albuquerque, New Mexico, USA) at the 50% anterior mandibular position as the initial position, as measured with a George Gauge (George 1992). Either a standard size or a large size was applied, depending on the width of the dental arch. TheraSnore^TM^ appliance is a boil‐and‐bite appliance with two pieces; the lower section is adjustable forward and backward in 1.5‐mm increments (maximum, 3 mm). The device consists of two trays of semi‐rigid thermoplastic material supported by a framework of harder, heat‐resistant polycarbonate (Tsuda et al. 2010). Some mandibular movements are allowed, with the exception of backward movement during sleep with the device. If subjective improvement were not present, the jaw positions were advanced more forward (1.5 mm or 3.0 mm) without discomfort and/or pain in the temporomandibular joint (TMJ) area by patients and/or clinicians. All patients were instructed to bring the posterior teeth back into contact, to massage the temporal and masseter areas and TMJ area after using the OA. Measurements of overjet and overbite and palpation of the TMJ and masticatory muscles were performed, and no changes of them were observed every 2–3 weeks. If the patients suffered pain in the soft tissues or teeth in direct contact with the device, the devices was adjusted by clinicians until problems with OA use were resolved.

### Questionnaire

The originally designed, self‐reported questionnaire that was mailed to each patient was created using selected questions from previous studies (Almeida et al. 2005; Clark et al. 2000; Jauhar et al. 2008; Pantin et al. 1999) and our clinical experiences. A reply‐paid envelope was included. All patients were asked whether they were using the OA and classified into two groups based on the response: using group and not‐using group. If the patient answered “yes”, they were asked about the frequency of use (almost every night, 3–4 times/week, 1–2 times/week, or 1–2 times/month), setting position (3 mm forward; 1.5 mm forward; no change; 1.5 mm backward; 3 mm backward), improved signs and subjective symptoms (multiple answers allowed), and side effects of the OA (multiple answers allowed). If they answered “no”, they were asked why they quit using the OA (multiple answers allowed) and when they quit using it.

### Overnight polysomnography

All patients were provided with letters of introduction to the medical institution at which they had been diagnosed with OSA for overnight PSG 3 or 4 months after starting OA treatment. At the time of this survey, 12 patients in the using group and seven patients in the non‐using group underwent PSG with the OA in place. Medical doctors assessed their data again. Apnea index (AI), apnea‐hypopnea index (AHI), minimum hemoglobin oxygen saturation measured by pulse oximetry (minimum SpO_2_), and arousal index from overnight PSG data before and after treatment were used to evaluate treatment effects of the OA. In addition, treatment efficacy was evaluated based on goals of AHI < 10/h and a percentage reduction in AHI of more than 50% compared with baseline (Almeida et al. 2009; Krishnan et al. 2008; Kuna et al. 2006).

### Statistical analysis

spss for Macintosh version 22.0 software (SPSS, Chicago, IL, USA) was used to analyze the data. Data are presented as mean ± standard deviation. To compare characteristics between the using group and not‐using group, the unpaired Student's *t*‐test, Mann–Whitney test, and Pearson's chi‐squared test were used. To assess the statistical significance of differences before and after treatment in compliance and not‐using groups, the paired Student's *t*‐test was used. To compare the treatment efficacy between the using and not‐using groups, Pearson's chi‐squared test was used. Values of *p* < 0.05 were considered significant.

## Results

Completed questionnaires were received from 48 patients (response rate, 36%). Four patients were not able to be contacted because of missing address information (*n* = 3) and death (*n* = 1). Of the 48 responding patients, 33 (68.8%) were still using the OA (using group). No significant differences in baseline data were seen between the using and not‐using groups (Table 1).

**Table 1:** Characteristics of compliance and not‐using groups

Characteristics	Using group (*n* = 33)	Not‐using group (*n* = 15)	*P*
Age, years	63.3±12.41	66.5±11.84	0.40661^a^
Gender (M/F), *n*	24/9	11/4	0.75917^b^
BMI (kg/m^2^)	23.6±2.99	23.7±2.53	0.92331^a^
Baseline AI	12.3±13.30	11.6±9.70	0.64660^c^
Baseline AHI	23.4±15.73	20.0±10.55	0.59338^c^
Baseline minimum oxygen saturation	79.1±12.01	81.9±6.05	0.91197^c^
Baseline arousal index	17.6±10.83	20.1±10.70	0.50498^a^

Data are presented as mean±SD or number.

a Unpaired Student's *t*‐test

b Chi‐square test

c Mann–Whitney *U* test

In the using group, mean duration of OA use was 18.8 ± 23.4 months. Device use was “almost every night” in 19 patients (57.6%), “3–4 times/week” in 5 patients (15.2%), “1–2 times/week” in 3 patients (9.1%), and “1–2 times/month” in 6 patients (18.2%).

The OA was being used at the 50% anterior mandibular position in 17 patients (51.5%), 3.0 mm forward in seven patients (21.2%), 1.5 mm forward in seven patients (21.2%), 1.5 mm backward in 1 patient (3.0%), and 3.0 mm backward in 1 patient (3.0%).

Thirty patients (90.9%) showed improvement in signs and subjective symptoms, while two patients (6.1%) reported no improvement, and one patient (3.0%) provided no answer. Of these 30 patients, seven patients (23.3%) were using the device at 3.0 mm forward, six (20.0%) at 1.5 mm forward, 15 (50.0%) at the 50% anterior mandibular position, and one each (3.3%) at the 1.5 mm backward and 3.0 mm backward positions.

**Figure 1:**
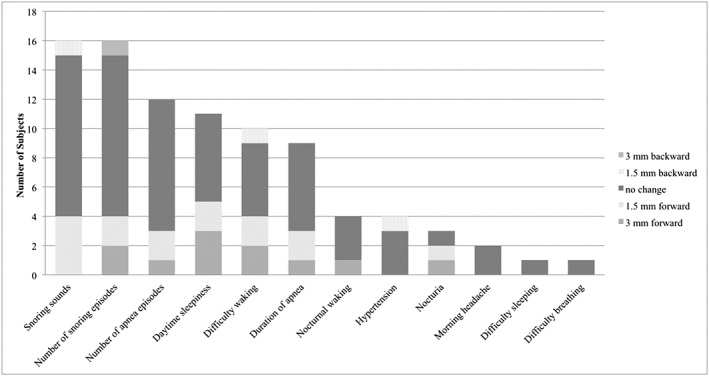
Improved signs and subjective symptoms (multiple answers allowed) (*n* = 33).

Improved signs and subjective symptoms were “snoring sounds”, “number of snoring episodes” (*n* = 16 each), “number of apnea episodes” (*n* = 12), “daytime sleepiness” (*n* = 11), “difficulty waking” (*n* = 10), “duration of apnea” (*n* = 9), “nocturnal waking” and “hypertension” (*n* = 4 each), “nocturia” (*n* = 3), “morning headache” (*n* = 2), and “difficulty sleeping” and “difficulty breathing” (*n* = 1 each) (Fig. 1).

**Figure 2:**
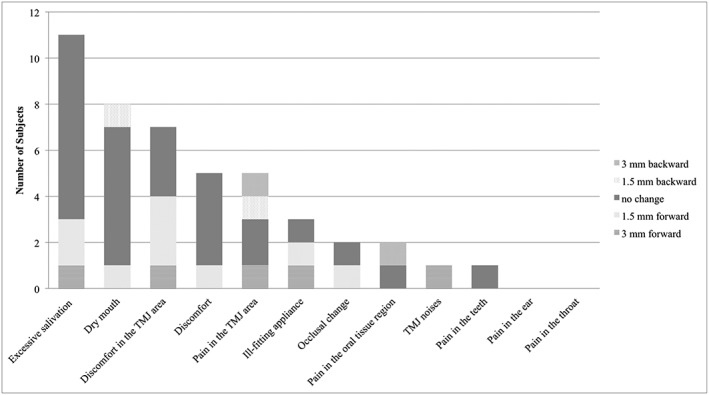
Side effects of oral appliances (multiple answers allowed) (*n* = 33).

Side effects were “excessive salivation” (*n* = 11), “dry mouth” (*n* = 8), “discomfort in the TMJ area” (*n* = 7), “discomfort” and “pain in the TMJ area” (*n* = 5 each), “ill‐fitting”(*n* = 3), “occlusal change” and “pain in the oral tissue region” (*n* = 2 each), and “TMJ noises” and “pain in the teeth” (*n* = 1 each). No compliant patient reported “pain in the ear” or “pain in the throat” (Fig. 2).

In the not‐using group, five patients (33.3%) quit using the OA within the first month, one patient (6.7%) at 1–3 months, one patient (6.7%) at 4–6 months, two patients (13.3%) at 7–12 months, and two patients (13.3%) after 12 months, while four patients (26.7%) did not provide an answer.

**Figure 3:**
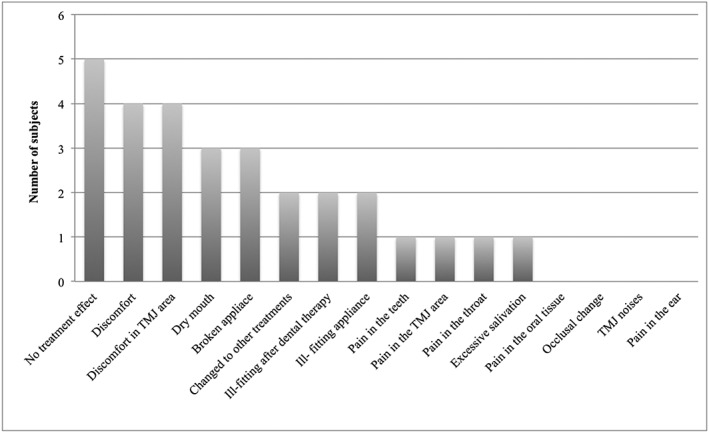
Reasons for quitting use of oral appliances (multiple answers allowed) (*n* = 15).

Of the 15 patients in the not‐using group, reasons for quitting use of the OA were “no treatment effect” (*n* = 5), “discomfort” and “discomfort in the TMJ area” (*n* = 4 each), “dry mouth” and “broken appliance” (*n* = 3 each), “changed for other treatment”, “ill‐fitting appliance after dental therapy” and “ill‐fitting appliance” (*n* = 2 each), “pain in the teeth”, “pain in the TMJ area”, “pain in the throat”, and “excessive salivation” (*n* = 1 each). No compliant patient reported “pain in the oral tissue region”, “occlusal change”, “TMJ noises”, or “pain in the ear” (Fig. 3).

In the using group, significant improvements were seen for AI, AHI, and minimum SpO_2_ (*p* = 0.0353, 0.0118, 0.0272). However, arousal index did not differ significantly between before and after treatment. In the not‐using group, no significant improvement was apparent in any indices (Table 2).

**Table 2:** Change of indices from polysomnography recording before and after treatment

Variable	Using group (*n* = 12)			Not‐using group (*n* = 7)		
	Pre‐treatment	Post‐treatment	*P*	Pre‐treatment	Post‐treatment	*P*
AI	18.6±16.77	7.2±13.36	0.03526*	9.7±11.00	1.5±1.18	0.15010
AHI	27.9±14.76	14.4±15.67	0.01180*	15.7±7.67	6.4±4.43	0.07434
minimum SpO_2_	78.3±11.07	83.2±6.97	0.02721*	85.9±3.85	88.1±6.47	0.41586
arousal index	18.1±10.67	13.5±10.83	0.32435	20.3±10.31	15.2±16.71	0.58794

Data presented as mean±SD or number.

Paired Student's *t*‐test was used.

* Significant difference (*p* < 0.05)

In the using group, eight out of 12 subjects (66.7%) were successfully treated, achieving an AHI < 10/h and >50% reduction in AHI. All eight patients were using OAs at the 50% anterior mandibular position. In the not‐using group, four out of seven subjects (57.1%) were successfully treated, achieving AHI < 10/h and >50% reduction in AHI. No significant difference in treatment efficacy was identified between the using and not‐using groups.

## Discussions

This questionnaire‐based study presented a response rate of 35.6%, lower than previous studies (Almeida et al. 2005; Cistulli et al. 2004). The questionnaire was sent only once in the present study, while most previous studies used telephone reminders or resent the questionnaire if the patient did not answer.

Few studies have reported that all compliant patients used the devices every night (Mehta et al. 2001; Pancer et al. 1999; Pantin et al. 1999). In the present study, the compliance rate was 68.8% (33/48), and among the 33 patients in the using group, 19 (57.6%) were using the devices “almost every night”, five (15.2%) “3–4 times/week”, three (9.1%) “1–2 times/week”, and six (18.2%) “1–2 times/month” after 18.8 months. The number of patients using the device every day was smaller than in previous studies (Ferguson et al. 1996, 1997; Johnston et al. 2002; McGown et al. 2001), but more than 70% of patients were using the device at least several times a week. Two of the six patients using the device 1–2 times a month were mainly using continuous positive airway pressure. These patients were using the device only when staying away from home.

In the present study, 30 patients (90.9%) showed improvements in signs and subjective symptoms. Common improved symptoms involved snoring, apnea, and daytime sleepiness, which are frequent symptoms of OSA. These results were similar to those reported in most studies (Bloch et al. 2002; Gotsopoulos et al. 2002). Dieltjens et al. (2012) suggested that the “target protrusion” was needed to be determined individually for every patient. In the present study, titration was based on subjective outcome without taking into account any objective parameters, which was based on the physical limits of the patients solely. Other forms of titration are controlled using PSG or some other objective measurement, such as home oximetry (Almeida et al. 2002), and are thus based on objective criteria alone. The best titration is multiparametoric, combining both the evolution of subjective symptoms and objective measurements, and this approach is currently used in screening and follow‐up assessment for better OA treatment success. PSG data was not obtained after treatment from all OSA patients, because PSG costs several hundred dollars in Japan.

Furthermore, Scherr et al. (2014) stated that OAs should allow the mandible to be advanced in increments of 1 mm or less with a protrusive adjustment range of at least 5 mm. TheraSnore^TM^ appliance is adjustable forward and backward in 1.5 mm increments, and thus cannot be strictly classified as a titratable appliance. The national insurance system permits a cost for delivery of OAs for OSAS of $227–270 in Japan, while the cost of customized, titratable OAs is $2,000–3,000 (Levendowski et al. 2012), so use of customized, titratable OAs for the first phase of OA therapy is difficult.

Larger mandibular protrusion will produce a larger decrease in OSA events (Clark et al. 1993; Ferguson *et al*. 2006), while some studies have shown a relationship with increased side effects such as occlusal changes including tooth pain (Aarab et al. 2010; Almeida et al. 2005; Marklund et al. 2001; Pantin et al. 1999), TMD (Cunali et al. 2009; Johnston et al. 2002) and increased protrusion. In the using group, no relationship was found between final mandibular position and numbers and/or types of side effects. Significant improvement in AI, AHI, and minimum SpO_2_ were found. Eight out of 12 subjects (66.7%) were successfully treated, achieving an AHI < 10/h and >50% reduction in AHI. All eight patients were using OAs at the 50% anterior mandibular position. Even though the mandibular protrusion distance was small, our treatment success rate was similar to those reported from previous studies (Almeida et al. 2009; Krishnan et al. 2008; Kuna et al. 2006). No patients were not obese (BMI < 25 in all patients) in the present study. These results suggest that the 50% anterior mandibular position is easy to accept while still offering some effects in OSA patients. Some OSA patients might acquire treatment efficacy, even though the mandibular protrusion is not large.

Lindman and Bondemark (2001) reported that a temporary bite change in the morning after removal of the OA occurs in almost all patients. In the present study, all patients were instructed to bring the posterior teeth back into contact and to massage the temporal and masseter areas and TMJ area after using the OA. Such practices might lead to minimization of onset bite change as a side effect. The two most frequent side effects have been reported as jaw or muscle pain and tooth pain (Clark et al. 2000; McGown et al. 2001), but excessive salivation and dry mouth were more common in the present study. This might be related to the shape and design of the TheraSnore™ appliance, a boil‐and‐bite appliance covering the maxillary teeth and oral mucosa with soft material. This device may thus have caused difficulty swallowing saliva. Three patients in the using group and two patients in the not‐using group reported “ill‐fitting appliance”. Deterioration of thermoplastic materials of the TheraSnore^TM^ appliance might have led to loose contact with teeth or gingival tissue. A systematic review of the evidence has shown that custom OAs are more effective than non‐custom OAs (Ramar et al. 2015). If the patients used custom OAs at the same mandibular protrusion, treatment efficacy would presumably have been greater than in our results.

In the not‐using group, one‐third of patients quit using the OA within the first month and 40% within the first 3 months. The most common self‐reported reasons for quitting use of the OA were a lack of treatment effect or discomfort or pain on use, similar to the previous study (Clark et al. 2000).

Marklund et al. (2001) reported that patients tended to quit using OAs due to not only side effects but also poor efficacy. The not‐using group showed no significant improvement in all indices, even though four out of seven subjects (57.1%) were successfully treated, achieving AHI < 10/h and >50% reduction in AHI. These results suggested that some patients would quit using OAs because wearing them is uncomfortable, despite achieving good efficacy. Vanderveken et al. (2008) showed significant differences in treatment effects between custom‐made and boil‐and‐bite devices. Therefore, custom‐made devices might provide better fit than boil‐and‐bite devices, which may in turn lead to increased compliance rates. If a patient using a boil‐and‐bite device fails to achieve satisfactory treatment effects at the first phase, the device should be changed to a custom‐made device or designs and materials of device should be improved.

## Conflicts of interest

None declared.
